# Inter-Species Interactions in the Fly Gut Microbiome Shape Aging

**DOI:** 10.1093/geroni/igaa057.2638

**Published:** 2020-12-16

**Authors:** William Ludington

**Affiliations:** Carnegie Institution, Baltimore, Maryland, United States

## Abstract

Gut bacteria affect key aspects of host fitness, including fecundity and lifespan. However, it is unclear to what extent individual species versus complex interactions drive host fitness. We dissected the natural microbiome of Drosophila melanogaster and revealed that interactions between bacteria shape host fitness through life history tradeoffs. Empirically, we made germ-free flies and colonized them with each possible combination of the five core species of bacteria. We measured the microbiome and fly fitness traits including reproduction and lifespan. Notably, flies that reproduced more died sooner. Removing bacteria after reproduction extended lifespan in most cases, suggesting an indirect tradeoff. However, in certain cases, antibiotics did not extend lifespan, indicating a metabolic memory of the microbiome. Overall, complex interactions within the microbiome had significant effects on host fitness. We suggest that model systems with reduced complexity will be instrumental in elucidating mechanisms of microbiome-host interactions.

